# Exploring the diagnostic and prognostic value of the C-reactive protein/lymphocyte ratio for dilated cardiomyopathy based on a real-world study

**DOI:** 10.1038/s41598-023-46338-y

**Published:** 2023-11-02

**Authors:** Bin Qi, Zhi-Jie Yang, Nan Huang, Wen-Bo Zheng, Chun Gui

**Affiliations:** 1 Department of Cardiology, The First Affiliated Hospital of Guangxi Medical University, Nanning, People’s Republic of China; 2 Guangxi Key Laboratory Base of Precision Medicine in Cardiocerebrovascular Diseases Control and Prevention, Nanning, People’s Republic of China; 3 Guangxi Clinical Research Center for Cardiocerebrovascular Diseases, Nanning, People’s Republic of China

**Keywords:** Biomarkers, Cardiology, Diseases, Risk factors

## Abstract

To determine the risk factors for dilated cardiomyopathy (DCM) and construct a risk model for predicting HF in patients with DCM, We enrolled a total of 2122 patients, excluding those who did not meet the requirements. A total of 913 patients were included in the analysis (611 males and 302 females) from October 2012 to May 2020, and data on demographic characteristics, blood biochemical markers, and cardiac ultrasound results were collected. Patients were strictly screened for DCM based on the diagnostic criteria. First, these patients were evaluated using propensity score matching (PSM). Next, unconditional logistic regression was used to assess HF risk. Furthermore, receiver operating characteristic (ROC) curve analysis was conducted to determine diagnostic efficiency, and a nomogram was developed to predict HF. Finally, the Kaplan‒Meier survival curve was plotted. Of the initial 2122 patients, the ejection fraction (EF) in males was worse. We included 913 patients after the final DCM diagnosis. The results showed that the levels of NT-proBNP, WBC, PLT, neutrophils, lymphocytes, eosinophils, and IL-6, C-reactive protein (CRP) and the neutrophil/lymphocyte ratio (NLR), platelet/lymphocyte ratio (PLR), and CRP/lymphocyte ratio (CLR) were higher in males than in females (*P* < 0.001–0.009). The nomogram showed that factors such as sex, WBC, neutrophils, PLR, and CLR could predict the risk of worsening cardiac function in patients with DCM before and after PSM (*P* < 0.05). The ROC curve showed that CLR with an 85.6% area demonstrated higher diagnostic efficacy than the NLR (77.0%) and PLR (76.6%, *P* < 0.05). Survival analysis showed a higher mortality risk in females with higher CLR levels (*P* < 0.001–0.009). However, high CLR levels indicated a higher mortality risk (*P* < 0.001) compared to sex. Male EF is lower in DCM patients. CLR could predict the risk of declined cardiac function in patients with DCM. The mortality in females with higher CLR levels was highest; however, the exact mechanism should be investigated.

## Introduction

Dilated cardiomyopathy (DCM) is a heterogeneous condition associated with genetic abnormalities, viral infection, myocardial inflammation, and other etiological factors. However, the pathogenesis of DCM is still unclear^1^. DCM is the major cause of heart failure (HF) and is often the development and outcome of heart transplantation. The overall risk of HF in males and females is similar; however, there is a significant difference in HF risk based on sex, which is overlooked^2^.

Several studies have explored the involvement of persistent inflammation in the pathogenesis of DCM^3^. An increase in the expression level of inflammatory factors, such as C-reactive protein (CRP) and IL-6, was observed in patients with DCM, which correlated with the severity of cardiac function impairment. Women are more susceptible to endothelial inflammation, which indicates differences in immune responses based on sex. The levels of proinflammatory cytokines and markers, such as CRP, proinflammatory T-cell activation, and overall inflammation, are high in females^4^. A study showed an increase in proinflammatory gene expression levels in the myocardium of females compared to males^5^. These data indicate that the incidence of DCM in females could be higher, and the prognosis of DCM in females could be poor. However, the morbidity and mortality in males with heart disease are higher^1^. Therefore, additional studies are needed to determine the difference in the risk of DCM based on sex and identify reliable indicators to evaluate the correlation between immunity and the prognosis of DCM.

Studies have identified several novel hematological indicators, including the neutrophil/lymphocyte ratio (NLR), platelet/lymphocyte ratio (PLR) and CRP/lymphocyte ratio (CLR), which can steadily reflect the state of inflammation. The NLR and PLR are closely correlated with the pathogenesis of hypertension, coronary heart disease severity, the prognosis of chronic HF, and other cardiovascular diseases (CVDs)^6, 7^. The CLR response is closely related to the activity of inflammation^8^. In fact, few studies have determined the association among the NLR, PLR, CLR and DCM.

In this study, we analyzed the changes in NLR, PLR and CLR in patients with DCM and determined the significance of the NLR, PLR and CLR in predicting the prognosis of DCM. These results could aid in providing a reference for assessing mortality risk among patients with DCM.

Randomized controlled trials (RCTs) primarily follow a randomized and double-blinded approach, emphasizing standardized treatment. The disadvantages of RCTs include a short observation time for selected groups and a small sample size, which makes the implementation of RCTs challenging. The Real World Study (RWS) primarily evaluates medical events from the perspective of patients. The main concern of RSW is the effect. Our study is nonrandomized and nondouble-blinded in nature, which is similar to real-world medical environments and treatment approaches^9^. Observing the efficacy of the treatment approaches in a clinical setting would aid in determining the influence of gender differences and immune response on the prognosis of DCM.

## Results

### General clinical features of patients hospitalized for HF

**Figure 1:**
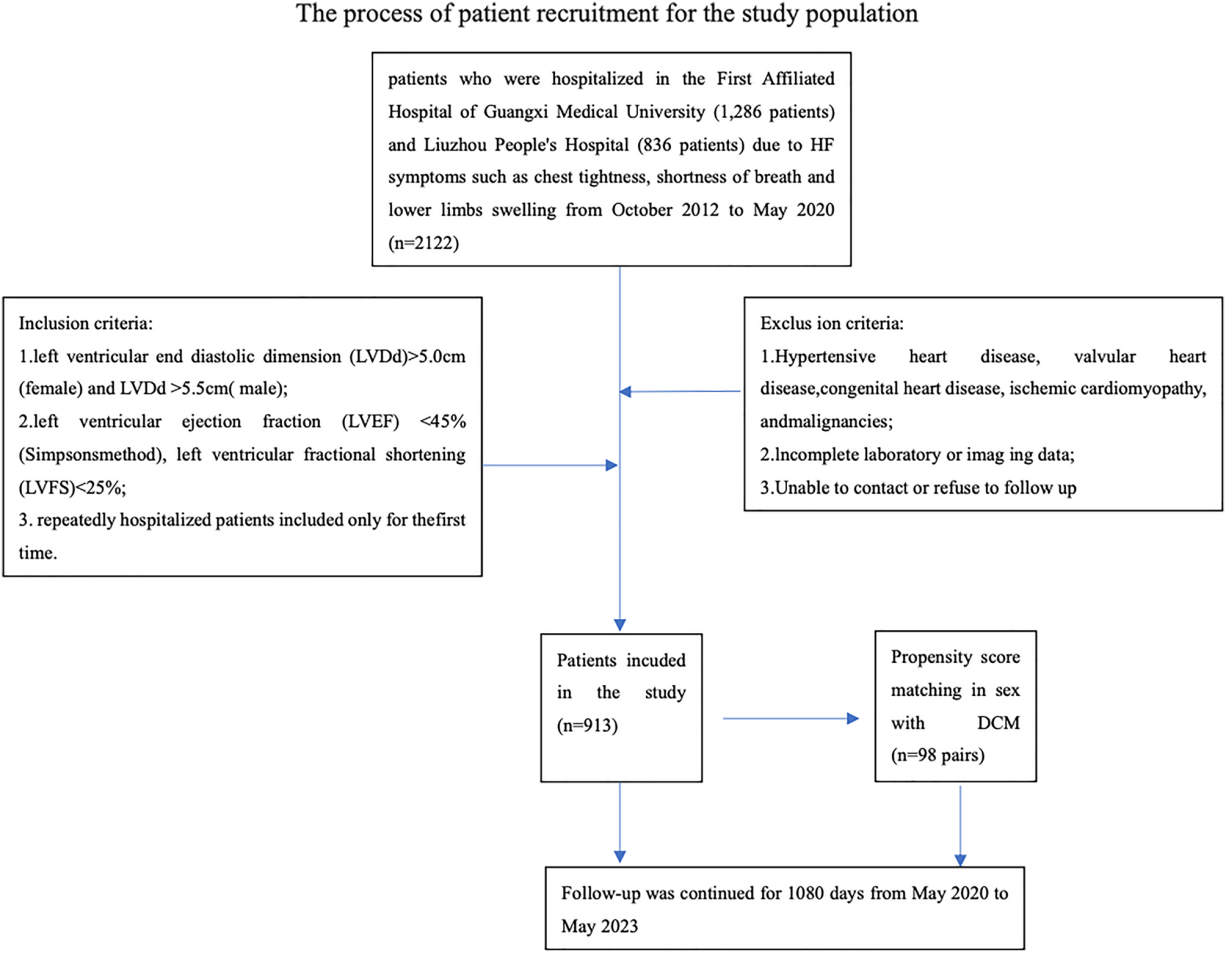
Process of recruiting patients for the study. HF, heart failure; DCM, dilated cardiomyopathy.

We divided 2122 patients into three groups based on EF (Fig. 1). Significant differences in age, sex, history of diabetes and coronary artery disease (CAD), educational status, DBP, SBP, and heart rate (HR) were observed among patients with different heart functions (*P* < 0.001, Table 1). Both in the general population of HF and in the specific ejection fraction subgroup, the proportion of males was higher than that of females.

**Table 1:** General information on patients with heart failure.

Variables	40 < EF < 45 (n = 512)	30 < EF < 40 (n = 986)	EF < 30 (n = 624)	F/χ^2^	*P*
Age (years)	59.22 ± 13.43	61.52 ± 12.42	64.87 ± 13.34	27.81	< 0.001
Male (n%)	306 (59.77)	622 (63.08)	444 (71.15)	17.95	< 0.001
BMI	25.54 ± 3.52	25.72 ± 2.76	25.46 ± 2.82	1.594	0.203
HBP (n%)	179 (34.96)	355 (36.00)	237 (37.98)	1.195	0.55
HLP (n%)	128 (25.00)	237 (24.04)	150 (24.04)	0.196	0.907
DM (n%)	87 (16.99)	128 (12.98)	56 (14.21)	16.30	< 0.001
CAD (n%)	134 (26.17)	384 (38.95)	274 (43.91)	39.90	< 0.001
AID (n%)	34 (6.64)	77 (7.81)	59 (9.46)	3.125	0.21
Cancer (n%)	6 (1.17)	17 (1.72)	17 (2.72)	3.923	0.141
High school or above (n%)	251(49.02)	414 (41.99)	220 (35.26)	21.98	< 0.001
SBP (mmHg)	127.35 ± 22.43	126.49 ± 23.56	123.37 ± 21.42	5.234	0.005
DBP (mmHg)	88.57 ± 11.22	85.30 ± 12.18	83.53 ± 10.69	27.37	< 0.001
HR (bpm)	73.57 ± 12.37	77.89 ± 10.69	79.31 ± 12.22	37.28	< 0.001

*BMI* body mass index; *HBP* high blood pressure; *HLP* hyperlipidemia; *DM* diabetes; *CAD* coronary artery disease; *AID* autoimmune diseases; *SBP* systolic blood pressure; *DBP* diastolic blood pressure; *HR* heart rate.

### Comparing the clinical data of patients with DCM

Patients with DCM were included based on the diagnostic, inclusion, and exclusion criteria, thorough examination, exclusion of other diseases, and ethical review approval. We enrolled 615 patients, including 414 males and 201 females, from the First Affiliated Hospital of Guangxi Medical University and 298 patients, including 197 males and 101 females, from Liuzhou People’s Hospital Affiliated with Guangxi Medical University (Table 2). The laboratory parameters and clinical features were compared between male and female patients with DCM. The results revealed that compared to females, age, BMI, history of smoking and alcohol consumption, SBP, DBP, LVEDD, LVESD, CK, CK-MB, NT-proBNP, LDL, FPG, WBC, PLT, neutrophils, lymphocytes, eosinophils, IL-6, and CRP were higher in males (*P* < 0.001–0.009). In addition, the EF of males was lower than that of females. (*P* < 0.001). Next, we calculated the NLR, PLR, CLR, CRP/WBC ratio (CWR), IL-6/WBC ratio (IWR), and IL-6/lymphocyte ratio (ILR). The results revealed that NLR, PLR, CLR, CWR, IWR, and ILR were higher in males than in females (*P* < 0.001–0.046).

**Table 2:** Clinical data of patients with dilated cardiomyopathy.

	Male (n = 611)	Female (n = 302)	t	*P*
General case				
Age (years)	52.68 ± 12.67	47.32 ± 12.13	6.099	< 0.001
BMI (kg/m^2^)	25.32 ± 2.32	24.34 ± 2.14	6.159	< 0.001
Life style				
Smoking (n(%))	147(24.06)	39(19.31)	15.48	< 0.001
Drinking (n(%))	102(16.69)	21(10.40)	16.45	< 0.001
Cardiac ultrasound				
LVESD	54.72 ± 4.37	52.25 ± 5.11	7.588	< 0.001
LVEDD	57.36 ± 4.74	55.19 ± 5.36	6.228	< 0.001
EF (%)	36.36 ± 2.87	39.72 ± 2.91	16.57	< 0.001
Vital signs				
S BP (mmHg)	131.24 ± 8.14	121.64 ± 6.86	17.63	< 0.001
DBP (mmHg)	79.23 ± 5.42	72.63 ± 5.21	17.53	< 0.001
HR (bpm)	76.13 ± 5.32	75.72 ± 5.41	1.089	0.276
Laboratory examination				
CK	223.72 ± 25.81	218.72 ± 24.93	2.785	0.006
CK-MB	18.23 ± 4.01	19.01 ± 4.22	2.717	0.007
NT-proBNP (Pg/mL)	1257.75 ± 117.32	1163.81 ± 102.13	11.87	< 0.001
Lipid				
TC	4.79 ± 1.01	4.72 ± 1.23	0.915	0.36
HDL	1.29 ± 0.37	1.27 ± 0.33	0.796	0.426
LDL	2.54 ± 0.82	2.28 ± 1.23	3.792	0.002
TG	1.21 ± 0.78	1.19 ± 0.81	0.36	0.719
FBG	5.45 ± 0.67	5.33 ± 0.61	2.621	0.009
HbA1c	6.02 ± 0.97	5.95 ± 0.86	1.064	0.288
blood routine examination				
WBC	8.32 ± 1.53	7.64 ± 1.21	6.75	< 0.001
RBC	117 ± 15.72	115 ± 16.54	1.778	0.076
PLT	233.40 ± 26.72	192.82 ± 23.90	22.34	< 0.001
Lymphocyte	6.12 ± 1.35	5.53 ± 1.17	6.485	< 0.001
Neutrophil	1.93 ± 0.47	1.57 ± 0.51	10.58	< 0.001
Monocyte	0.53 ± 0.21	0.52 ± 0.23	0.656	0.512
Eosinophils	0.61 ± 0.39	0.53 ± 0.31	3.112	0.002
Basophilic	0.01 ± 0.01	0.01 ± 0.01	0.001	0.998
NLR	0.32 ± 0.10	0.28 ± 0.12	5.313	< 0.001
PLR	38.42 ± 12.27	34.92 ± 8.27	4.479	< 0.001
IL-6	175 ± 32.08	158 ± 27.41	7.894	< 0.001
CRP	13.76 ± 6.32	11.31 ± 5.59	5.721	< 0.001
CLR	4.92 ± 2.94	4.01 ± 2.47	4.631	< 0.001
CRP/WBC	2.95 ± 1.52	2.41 ± 1.73	4.821	< 0.001
IL-6/WBC	30.01 ± 14.93	27.86 ± 14.07	2.086	0.037
IL-6/Mon	41.92 ± 20.15	44.89 ± 23.09	1.995	0.046
ACEI/ARB (n(%))	470(76.92)	215(71.19)	3.543	0.059
Β (n(%))	303(49.59)	134(44.37)	2.207	0.137
MRA (n(%))	331(54.17)	172(56.95)	0.631	0.427

*BMI* body mass index; *LVESD* left ventricular end systolic diameter; *LVEDD* left ventricular end diastolic diameter; *SBP* systolic blood pressure; *DBP* diastolic blood pressure; *HR* heartbeat; *CK* creatine kinase; *CK-MB* creatine kinase-Mb; *HDL-C* high-density lipoprotein cholesterol; *LDL-C* low-density lipoprotein cholesterol; *TC* total cholesterol; *TG* triglyceride; *FBG* fasting blood glucose; *WBC* white blood cell; *RBC* red blood cell; *PLT* platelet count; *CRP* C-reactive protein; *NLR* neutrophil/lymphocyte ratio; *PLR* platelet/lymphocyte ratio; *CLR* C-reactive protein/lymphocyte ratio; *MRA* mineralocorticoid receipt antagonist; ACEI/ARB, β, MRA (n%) proportion of representative drug use.

**Figure 2:**
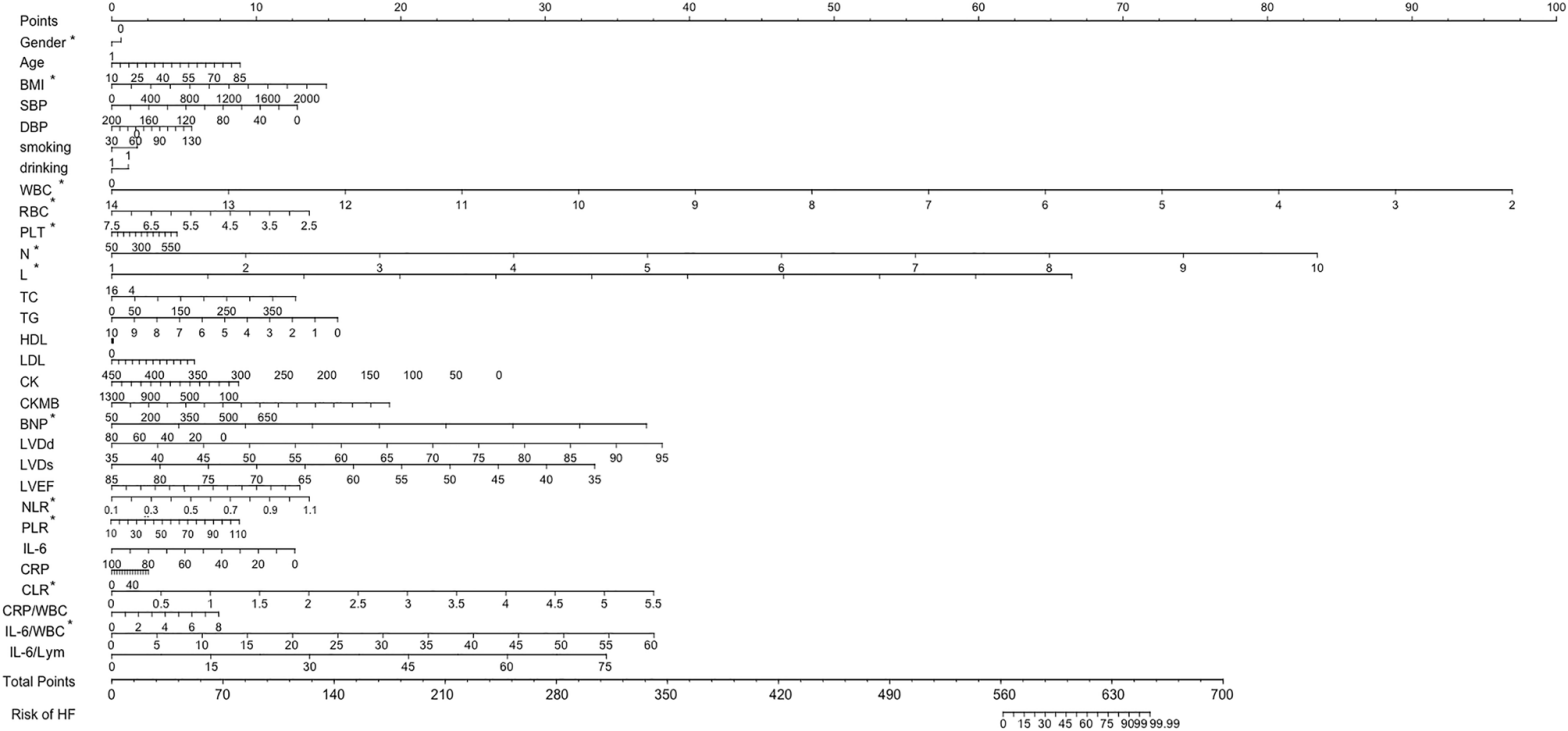
Nomogram for assessing the risk of HF in patients with DCM. HF, heart failure; DCM, dilated cardiomyopathy; BMI, Body Mass Index; DBP, diastolic blood pressure; SBP, systolic blood pressure; WBC, white blood cell counts; RBC, red blood cell counts; PLT, platelet counts; L, lymphocyte; N, neutrophil; TC, total cholesterol; TG, triglyceride; LDL, low-density lipoprotein; HDL, high-density lipoprotein; CK, creatine kinase; CK-MB, Creatine Kinase Isoenzyme; BNP, N-terminal B Type I natriuretic peptide; LVEDD, Left ventricular end-diastolic diameter; LVESD, left ventricular end-systolic diameter; LVEF, left ventricular ejection fraction; CRP, C-reactive protein; NLR, Neutrophil/lymphocyte ratio; PLR, platelet/ lymphocyte ratio; CLR, CRP/lymphocyte ratio.

Clinical and laboratory parameters, including sex, age, BMI, history of smoking and alcohol consumption, SBP, DBP, LVEDD, LVESD, EF, CK, CK-MB, NT-proBNP, LDL, FPG, WBC, PLT, neutrophils, lymphocytes, eosinophils, IL-6, and CRP, were included as risk factors to determine the declined cardiac function risk score and the risk model (nomogram, Fig. 2). The sores were defined as follows: smoking and drinking: yes = 1, no = 0, male = 0, and female = 1. The range of predicted probabilities of the declined cardiac function risk score was from 0 to 99.99%. The accuracy of the model in discriminating the patients was 0.822 (95% CI 0.743–0.886). The sensitivity and specificity of the model at an optimal cutoff value were 62.4% and 92.3%, respectively. The results revealed that the patient’s sex, BMI, NT-proBNP, WBC, RBC, PLT, neutrophils, lymphocytes, eosinophils, NLR, PLR, CLR, and IWR could predict the risk of cardiac function decline.

### PSM of patients with DCM

**Figure 3:**
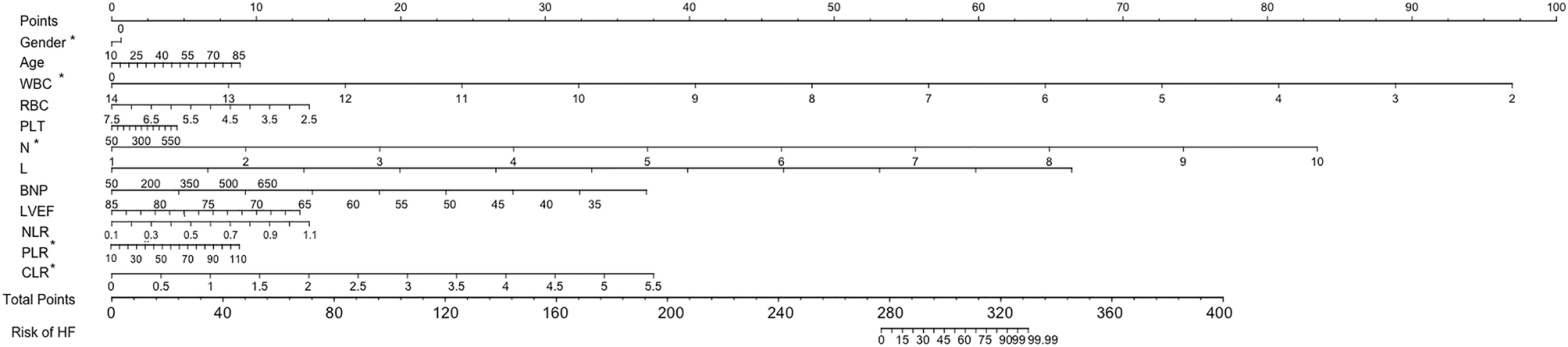
Nomogram for assessing the risk of HF in patients with DCM after PSM. HF, heart failure; DCM, dilated cardiomyopathy; WBC, white blood cells; RBC, red blood cells; PLT, platelet; L, lymphocyte; N, neutrophil; BNP, N-terminal B Type I natriuretic peptide; LVEF, left ventricular ejection fraction; CRP, C-reactive protein; NLR, Neutrophil /lymphocyte ratio; PLR, PLT/lymphocyte ratio; CLR, CRP/lymphocyte ratio.

In total 98 pairs of patients with DCM were enrolled after rigorous screening (Table 3). After PSM, the NT-proBNP, WBC, PLT, neutrophil, lymphocyte, and IL-6 levels, CRP, NLR, PLR, and CLR were higher in males than in females (*P* < 0.001). In patients with DCM before and after PSM, factors including patient sex, age, NT-proBNP, WBC, PLT, neutrophil lymphocytes, IL6, CRP, NLR, PLR, and CLR could reliably predict DCM risk in patients. The clinical and laboratory parameters, including sex, age, NT-proBNP, WBC, PLT, neutrophils, lymphocytes, IL-6, CRP, NLR, PLR, and CLR, could best predict the risk score of declined function and the risk model (nomogram, Fig. 3). We defined 0 and 1 as the sores for males and females, respectively. The range of predicted probabilities of cardiac function decline risk score was from 0 to 99.99%. The accuracy of the model in distinguishing the patients was 0.863 (95% CI 0.759–0.923). The sensitivity and specificity of the model at an optimal cutoff value were 68.7% and 94.8%, respectively. The results revealed that factors, including the patient’s sex, WBC, neutrophils, PLR, and CLR, could predict the risk of declined cardiac function. Moreover, both before and after PSM, factors including sex, WBC, neutrophils, PLR, and CLR could predict worsening cardiac function in patients with DCM.

**Table 3:** General information on propensity score matching patients with DCM.

Variables	Male (n = 98)	Female (n = 98)	t	*P*
LVESD	53.99 ± 4.41	53.89 ± 4.39	0.159	0.874
LVEDD	57.05 ± 4.71	56.99 ± 4.66	0.089	0.929
Vital signs				
SBP (mmHg)	125.13 ± 9.52	124.87 ± 9.63	0.19	0.849
DBP (mmHg)	83.27 ± 7.42	83.47 ± 7.47	0.188	0.851
HR (bpm)	76.56 ± 7.28	76.23 ± 7.19	0.319	0.75
Laboratory examination				
CK	221.71 ± 22.63	220.33 ± 22.81	0.422	0.673
CK-MB	18.19 ± 4.00	18.07 ± 4.03	0.287	0.774
NT-BNP (Pg/mL)	1244.63 ± 114.23	1161.49 ± 104.79	5.309	< 0.001
Lipid				
TC	4.71 ± 1.00	4.70 ± 1.03	0.069	0.945
HDL	1.31 ± 0.35	1.28 ± 0.33	0.617	0.538
LDL	2.45 ± 0.77	2.39 ± 0.79	0.538	0.591
TG	1.23 ± 0.75	1.21 ± 0.77	0.184	0.854
FBG	5.51 ± 0.66	5.49 ± 0.65	0.214	0.831
HbA1c	6.00 ± 0.91	5.99 ± 0.88	0.078	0.938
Blood routine examination				
WBC	8.53 ± 1.55	7.71 ± 1.17	4.18	< 0.001
RBC	116 ± 15.94	115 ± 16.05	0.438	0.662
PLT	241.73 ± 25.72	191.07 ± 21.12	15.07	< 0.001
Lymphocyte	6.93 ± 1.47	5.49 ± 1.73	6.279	< 0.001
Neutrophil	1.88 ± 0.51	1.50 ± 0.37	5.97	< 0.001
Monocyte	0.57 ± 0.23	0.56 ± 0.22	0.311	0.756
Eosinophils	0.59 ± 0.37	0.53 ± 0.30	1.247	0.214
Basophilic	0.01 ± 0.01	0.01 ± 0.01	0.001	0.998
NLR	0.34 ± 0.27	0.22 ± 0.19	3.598	< 0.001
PLR	36.43 ± 12.97	30.73 ± 9.31	0.186	< 0.001
IL-6	179 ± 32.36	160 ± 24.79	3.758	< 0.001
CRP	14.09 ± 6.21	11.55 ± 5.71	2.981	0.003
CLR	5.17 ± 2.73	4.47 ± 2.59	2.131	0.034
CWR	3.27 ± 1.71	2.79 ± 1.92	1.848	0.066
IWR	33.90 ± 15.77	29.91 ± 14.99	1.851	0.071
ILR	44.08 ± 21.92	43.32 ± 23.75	0.233	0.816
ACEI/ARB (n(%))	77(78.57)	72(73.47)	0.7	0.403
Β (n(%))	49(50.00)	44(44.90)	0.512	0.476
MRA (n(%))	55(56.12)	57(58.16)	0.083	0.773

*BMI* body mass index; *LVESD* left ventricular end systolic diameter; *LVEDD* left ventricular end diastolic diameter; *SBP* systolic blood pressure; *DBP* diastolic blood pressure; *HR* heartbeat; *CK* creatine kinase; *CK-MB* creatine kinase-Mb; *HDL-C* high-density lipoprotein cholesterol; *LDL-C* low-density lipoprotein cholesterol; *TC* total cholesterol; *TG* triglyceride; *FBG* fasting blood glucose; *WBC* white blood cell; *RBC* red blood cell; *PLT* platelet count; *CRP* C-reactive protein; *NLR* neutrophil/lymphocyte ratio; *PLR* platelet/lymphocyte ratio; *CLR* C-reactive protein/lymphocyte ratio; *MRA* mineralocorticoid receipt antagonist; ACEI/ARB, β, MRA (n%) proportion of representative drug use.

### Diagnostic efficiency

**Figure 4:**
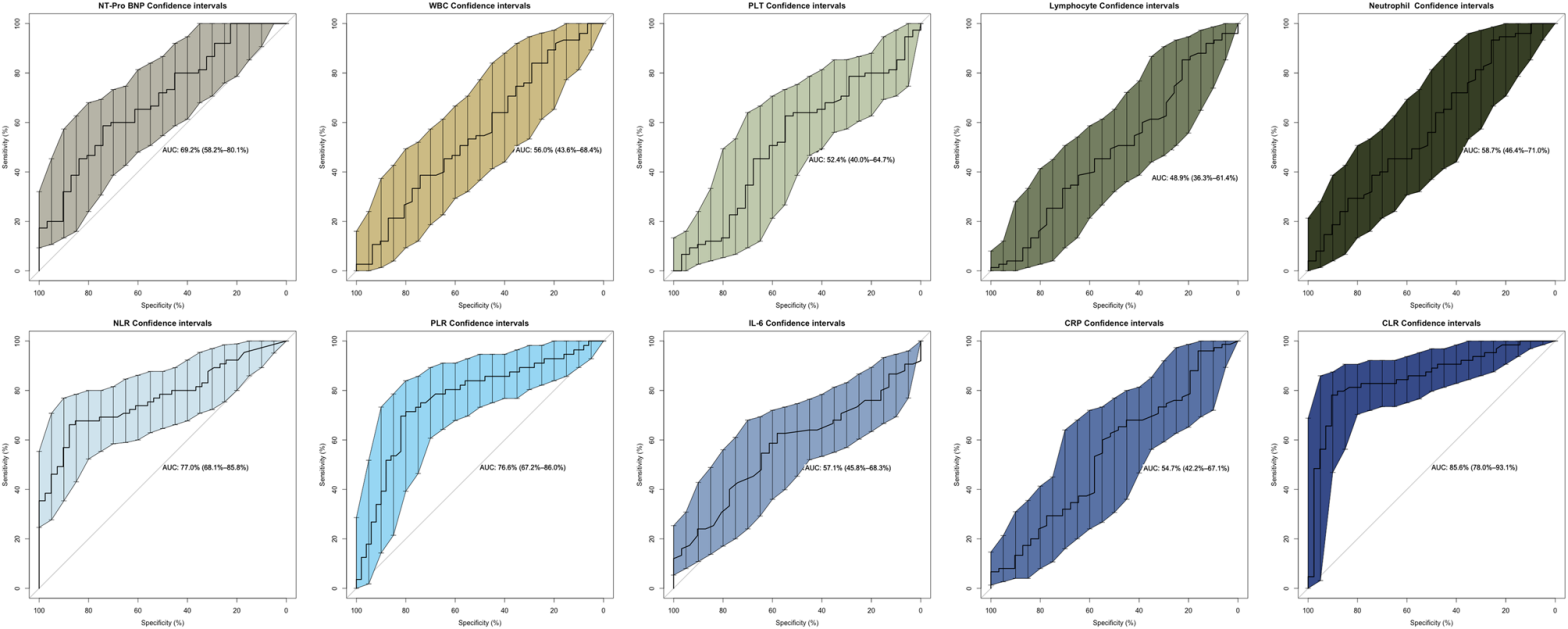
ROC curve for predicting severe HF in patients with DCM. HF, heart failure; DCM, dilated cardiomyopathy; WBC, white blood cell counts; RBC, red blood cell; PLT, platelet; L, lymphocyte; N, neutrophil; BNP, N-terminal B Type I natriuretic peptide; LVEF, left ventricular ejection fraction; CRP, C-reactive protein; NLR, Neutrophil /lymphocyte ratio; PLR, PLT/lymphocyte ratio; CLR, CRP/lymphocyte ratio.

**Figure 5:**
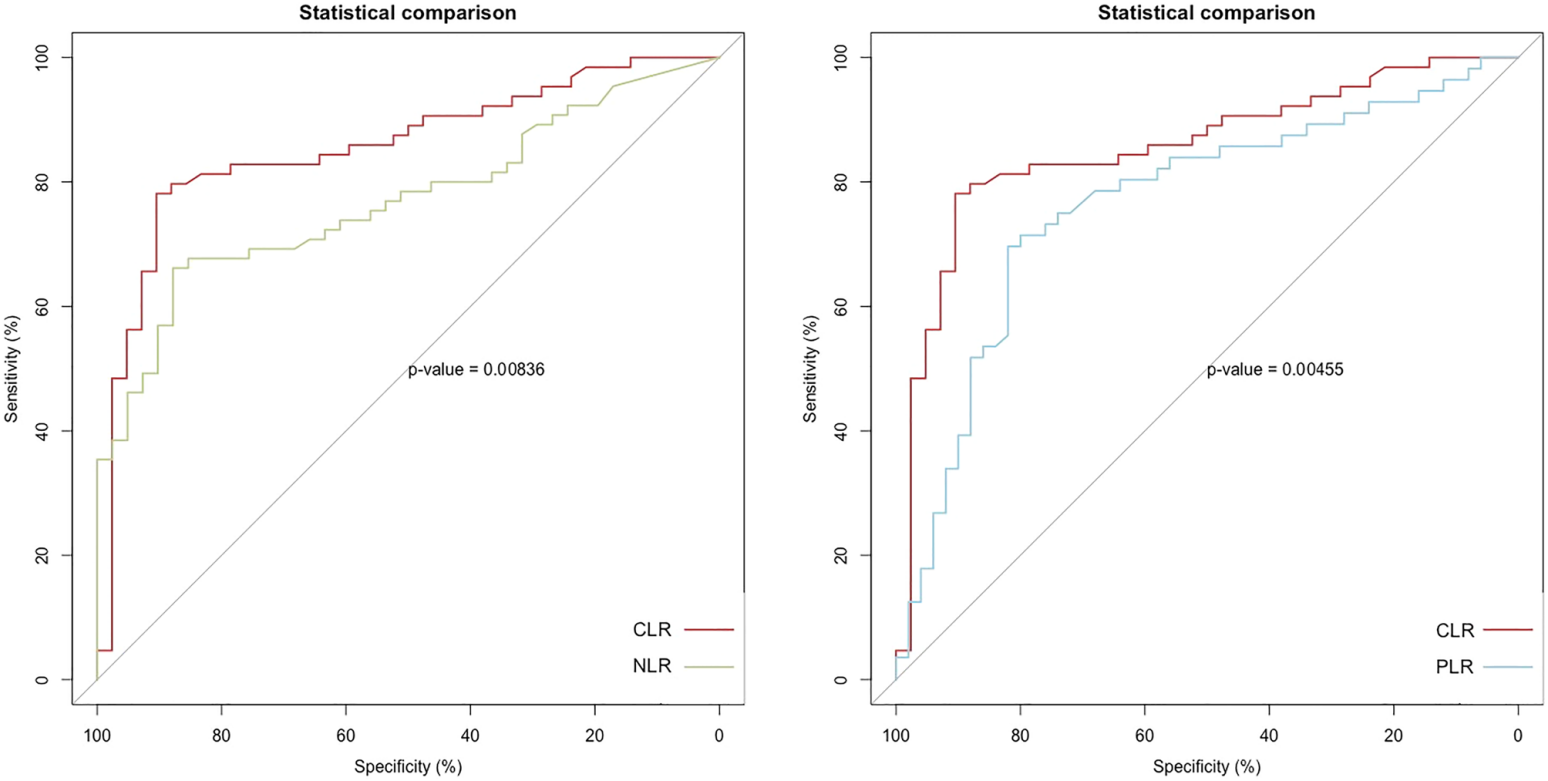
ROC curve shows a comparison of CLR with NLR and PLR. NLR, Neutrophil/lymphocyte ratio; PLR, platelet/lymphocyte ratio; CLR, CRP/lymphocyte ratio.

The results revealed that ten risk factors, including NT-proBNP, WBC, PLT, neutrophils, lymphocytes, IL-6, CRP, NLR, PLR, and CLR, were associated with changes in cardiac function for predicting DCM using PSM. The efficacy of these ten risk factors in predicting HF is shown in Fig. 4. Next, we calculated the AUC values to determine the diagnostic effectiveness of the risk factors as follows: CLR (0.856, 95% CI 0.780–0.931), NLR (0.770, 95% CI 0.681–0.858), PLR (0.766, 95% CI 0.672–0.860), NT-proBNP (0.692, 95% CI 0.582–0.801), neutrophils (0.587, 95% CI 0.464–0.710), IL6 (0.571, 95% CI 0.458–0.683), WBC (0.550, 95% CI 0.436–0.684), CRP (0.547, 95% CI 0.422–0.671), PLT (0.524, 95% CI 0.400–0.647), and lymphocytes (0.489, 95% CI 0.363–0.614). In conclusion, CLR demonstrated excellent diagnostic efficacy in predicting declined cardiac function. Moreover, the diagnostic efficacy of CLR was better than that of NLR (*P* = 0.008) and PLR (*P* = 0.005, Fig. 5).

### Follow-up of patients with DCM

**Figure 6:**
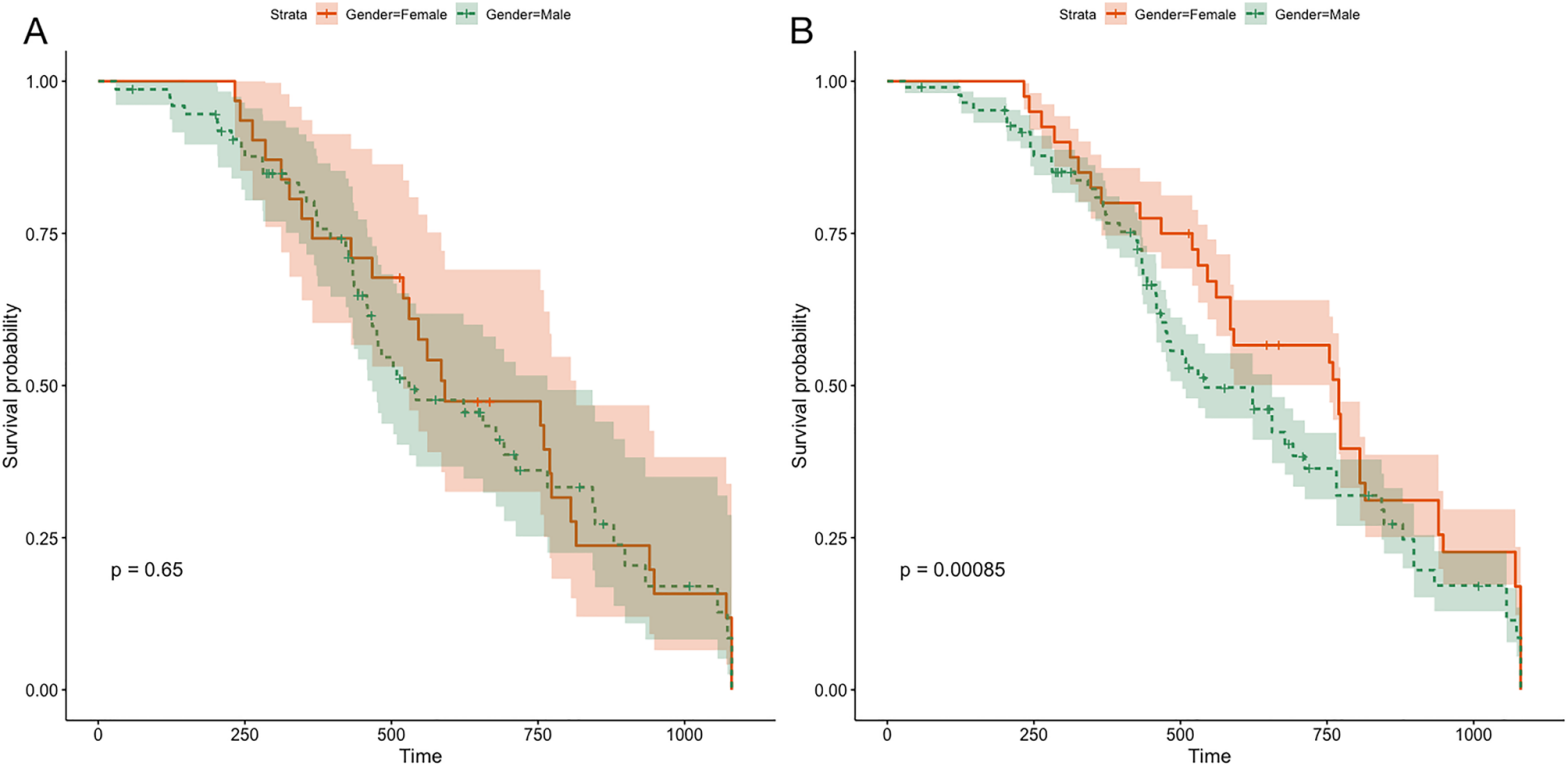
The KM survival curve shows the endpoint events in males and females with DCM during follow-up. (**A**) 913 patients (611 males and 302 females) with DCM. (**B**) 98 pair of patients with DCM after PSM. DCM, dilated cardiomyopathy.

**Figure 7:**
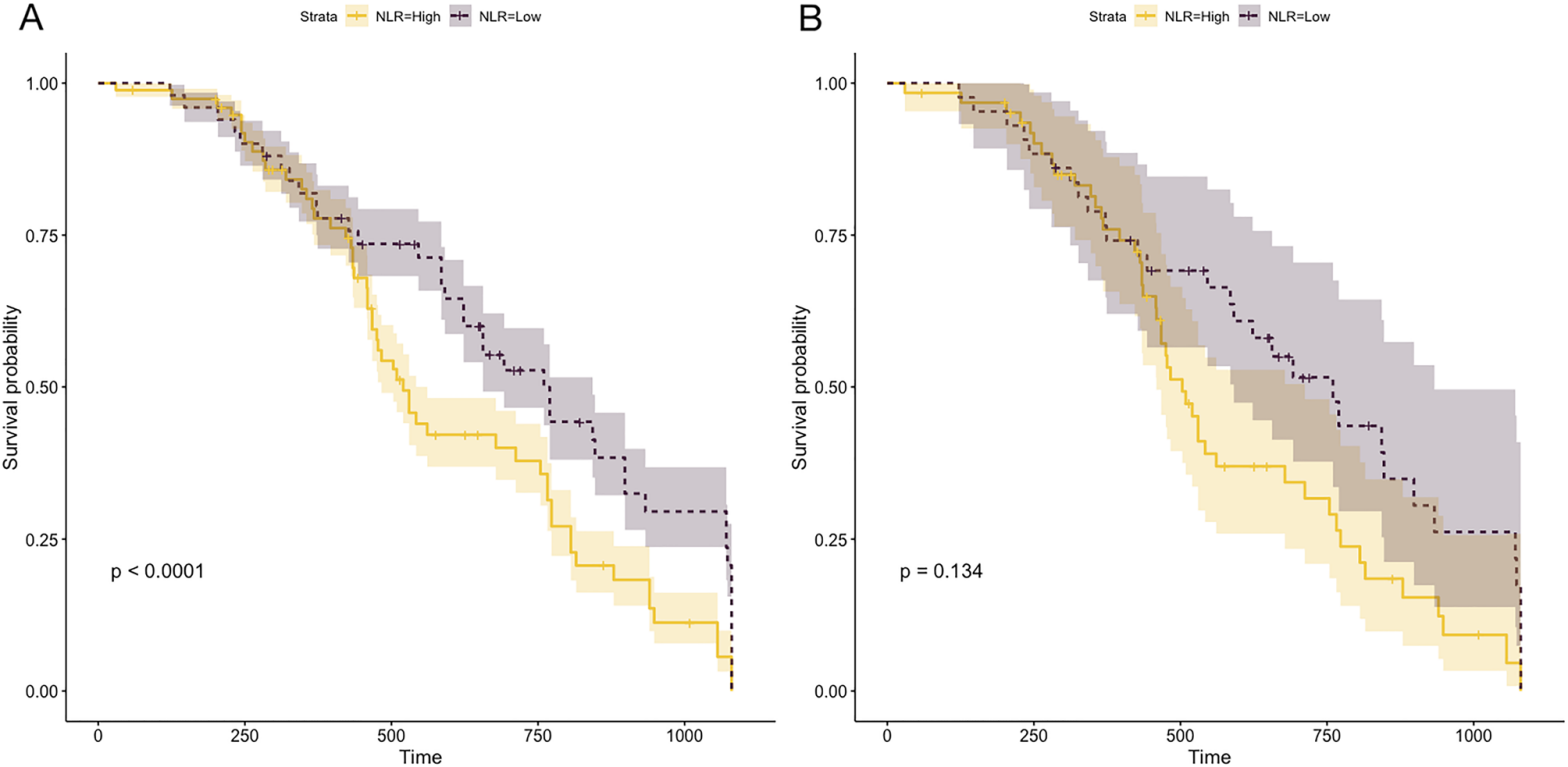
KM survival curve shows the endpoint events among DCM with high and low NRL levels during follow-up. (**A**) 913 patients (611 males and 302 females) with DCM. (**B**) 98 pairs of patients with DCM after PSM. NLR: neutrophil/lymphocyte ratio; DCM, dilated cardiomyopathy.

**Figure 8:**
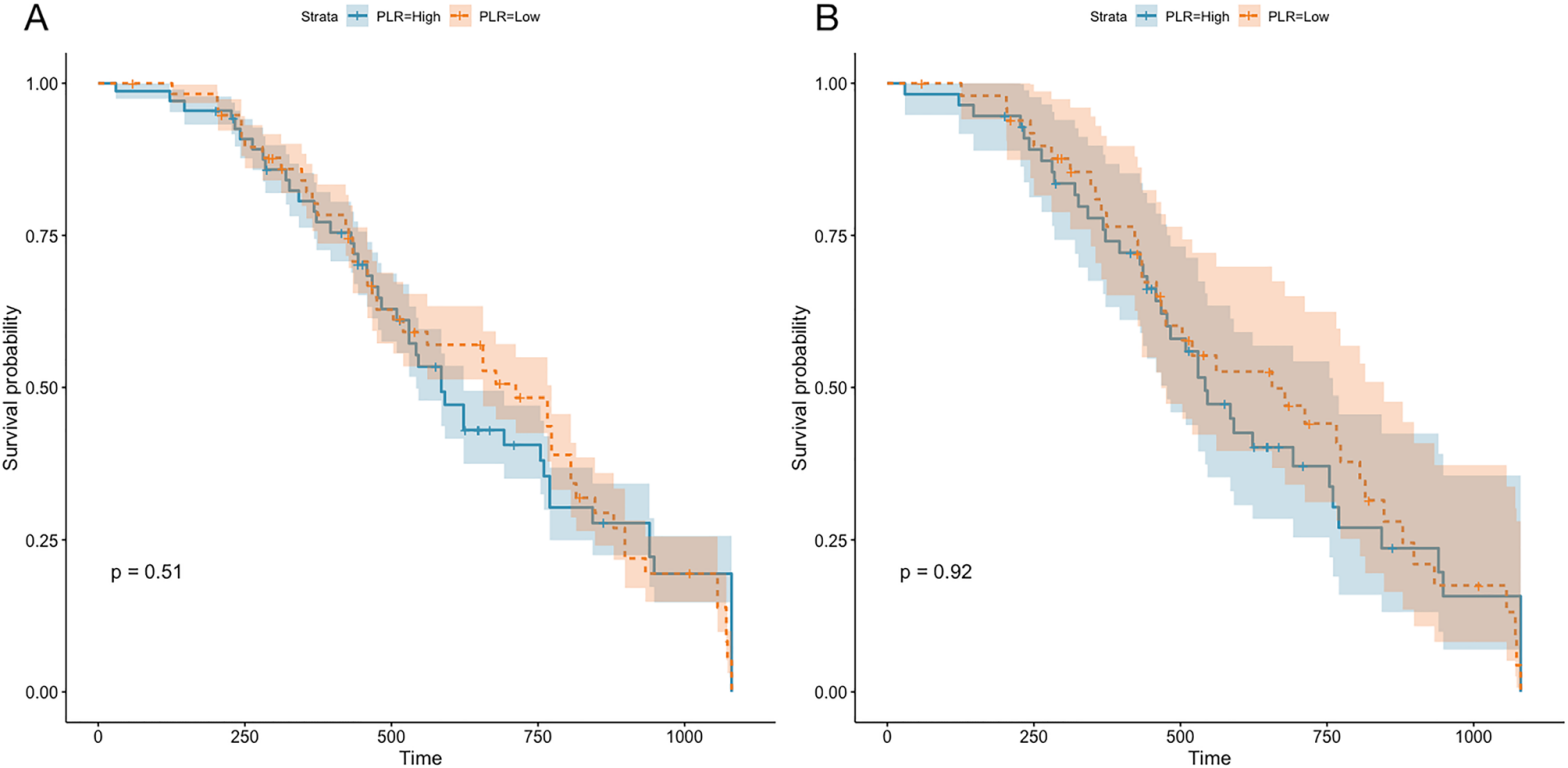
KM survival curve shows the endpoint events among DCM patients with high and low PRL levels during follow-up. (**A**) 913 patients (611 males and 302 females) with DCM. (**B**) 98 pairs of patients with DCM after PSM. DCM, dilated cardiomyopathy; PLR, platelet/lymphocyte ratio.

**Figure 9:**
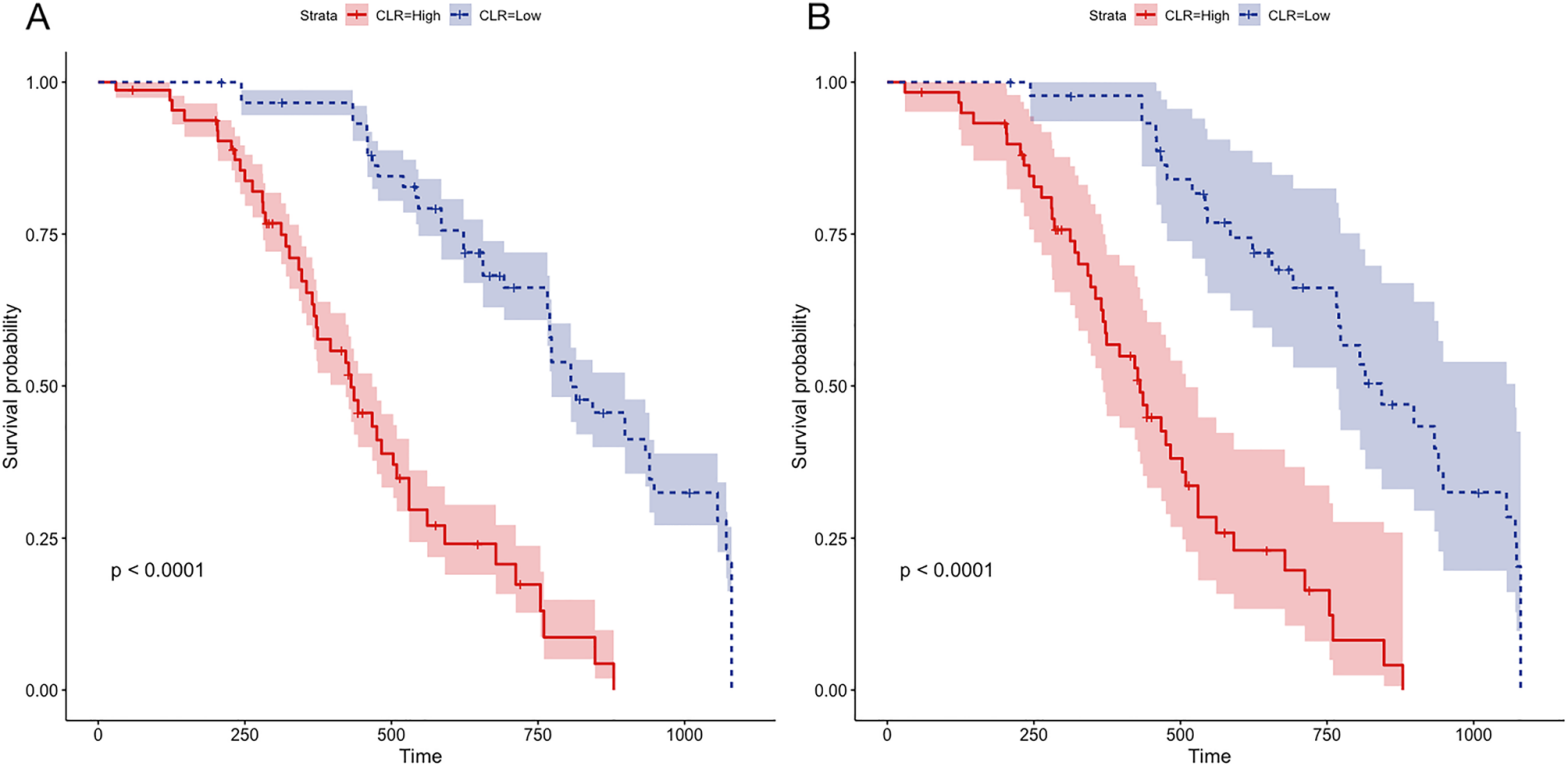
KM survival curve shows the endpoint events among DCM patients with high and low CRL levels during follow-up. (**A**) 913 patients (611 males and 302 females) with DCM. (**B**) 98 pair of patients with DCM after PSM. DCM, dilated cardiomyopathy; CLR, C-reactive protein/lymphocyte ratio.

**Figure 10:**
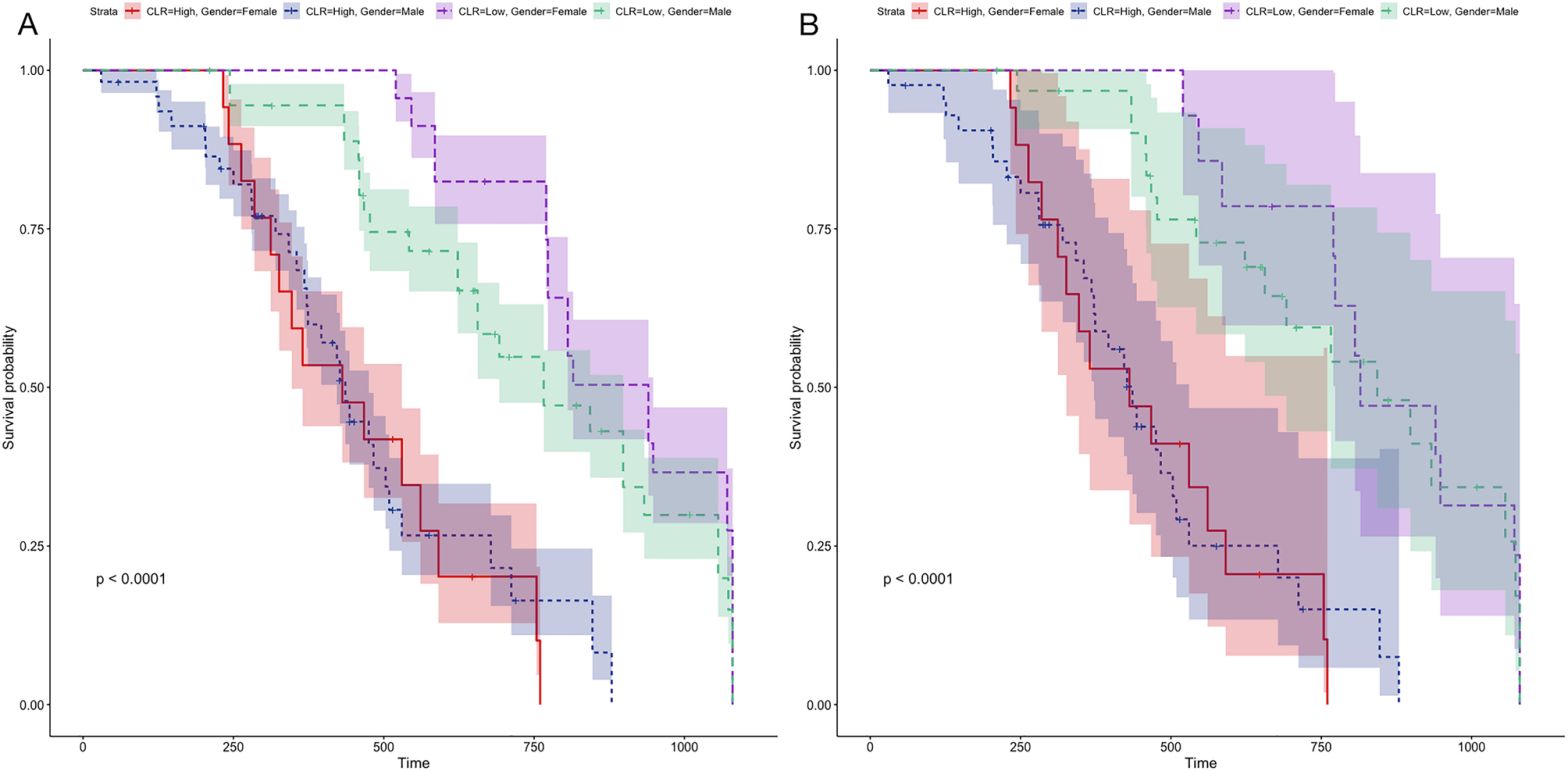
KM survival curve shows follow-up of patients with DCM. (**A**) 913 patients (611 males and 302 females) with DCM. (**B**) 98 pair of patients with DCM after PSM. DCM, dilated cardiomyopathy; CLR, C-reactive protein/lymphocyte ratio; NLR: neutrophil/lymphocyte ratio; PLR, platelet/lymphocyte ratio.

The endpoint of the follow-up was cardiogenic death and/or all-cause death. First, we divided 912 patients with DCM into two groups based on sex. The results revealed no significant difference in the mortality of patients based on sex after 1080 days of follow-up (*P* = 0.65, Fig. 6A). However, the prognosis of DCM in males was worse than that in females after PSM (*P* = 0.00085, Fig. 6B). Next, we divided 912 patients based on NLR levels and 1080 days of follow-up into two groups. The mortality of patients with a high NLR was high (*P* < 0.001, Fig. 7A); however, no significant difference was observed in patient mortality after PSM (*P* = 0.134, Fig. 7B). Furthermore, no significant difference in the risk of declined cardiac function was observed between patients with high PLR and those with low PLR in either the total DCM patients (*P* = 0.51) or in the PSM patients (Fig. 8). The survival rate of 912 patients with DCM with high CLR after PSM was the lowest (*P* < 0.001, Fig. 9). Finally, we comprehensively analyzed the effect of patient sex, CLR, NLR, and PLR on cardiac function. The results revealed that cardiac function declined quickly in females with high CLR, and their prognosis was poor among all patients with DCM or after PSM (*P* < 0.001, Fig. 10).

## Discussion

DCM is a specific type of cardiomyopathy caused by enlargement of the heart chambers for various reasons. DCM is a common cause of HF and death^10^. The onset of DCM is insidious in the early stage, and the prognosis of advanced-stage DCM is poor^11^. However, the diagnosis of DCM at an early stage and assessing the patient’s prognosis are challenging. In this study, we grouped patients with DCM based on sex to identify the factors predicting the differential risk of DCM in male and female patients. Next, we performed PSM and identified NT-proBNP, WBC, PLT, neutrophils, lymphocytes, IL-6, CRP, NLR, PLR, and CLR as risk factors for DCM. These factors were used to construct the HF risk model, and the results revealed that males of advanced age with high NT-proBNP, WBC, PLT, neutrophil, lymphocyte, IL-6, and CRP levels were at a higher risk of HF.

CRP is a common indicator of acute-phase heart disease. The liver produces CRP in response to IL-6 and is involved in pathophysiological functions during inflammatory processes. Patients with HF have high CRP levels associated with poor clinical outcomes^12, 13^. Activation of the immune response may play a role in HF by modifying renin–angiotensin–aldosterone and the sympathetic nervous system^14, 15^. Studies have shown that mononuclear macrophages and lymphocytes are chronically activated in patients with HF. CRP is a marker of chronic systemic inflammation and could be directly involved in congestive heart failure (CHF). CRP could induce the apoptosis of cardiomyocytes, thereby causing ventricular damage or dysfunction^16^. A study has demonstrated that CRP could independently predict the poor prognosis of DCM^17^. IL-6 is a proinflammatory cytokine primarily produced by activated monocytes and macrophages. The development of conditions such as cardiac arrest syndrome and heart failure is intricately connected to the pro-inflammatory characteristic of cytokines in the systemic inflammatory response^18, 19^. Previous studies have shown that IL-6 could predict the prognosis of CHF. A study has shown high IL-6 levels in most patients with DCM; high IL-6 levels were associated with low LVEF, atrial fibrillation, and poor clinical outcomes^20^. However, the correlation between platelets and the pathogenesis, as well as the prognosis of DCM, is still unclear. A study revealed that platelet-endothelial interactions could influence short- and long-term outcomes in patients with CHF^21^. While our results showed higher CRP, IL6, etc., in male patients than in females, this trend was still found after performing PSM, which is more in line with the findings of previous studies. These results are consistent with our HF risk model.

We used different biomarkers to determine the prognosis of DCM. Studies have identified NLR, PLR and CLR as new biomarkers of inflammation to predict the prognosis of patients with various diseases. Moreover, NLR and PLR were associated with higher mortality rates in children and adults with HF^22, 23^. A high NLR was observed in patients with poor functional grading, and a high NLR could predict mortality or heart transplantation in patients. A high NLR could predict a high rate of mortality in adults with decompensated HF compared to neutrophils, total WBC, and relatively low lymphocyte counts^22^. Moreover, the NLR could determine the severity of DCM in children and adults^23, 24^. Our results demonstrated that NLR and PLR could be used as indicators for assessing patients with DCM. Simultaneously, we identified CLR as a new marker with higher efficacy for diagnosing patients with DCM, and the diagnostic efficacy was even better than that of NLR and PLR. In DCM, the prognosis of patients with high CLR was poor. Few studies have used CLR to facilitate the diagnosis and predict the prognosis of HF or DCM. In fact, we showed that compared to the complexity of the risk model and the cost of monitoring NT-proBNP levels, CLR could be used as an efficient and inexpensive marker for assessing DCM. Studies have shown the involvement of CLR in evaluating coronavirus disease 2019^25^ and in predicting and evaluating postoperative patients with digestive cancer^26^. As the precise pathogenesis of DCM remains unclear, a recognized cause is persistent chronic inflammation, resulting in nonischemic necrosis of cardiomyocytes, one of the recognized etiologies is persistent chronic inflammation, leading to nonischemic necrosis of cardiomyocytes. Whereas CRP, neutrophils and lymphocytes are all responsive to the inflammatory response, NLR, PLR and CLR are all able to respond to the level of inflammation in the organism to some extent, which may be one of the reasons why CLR is able to predict the morbidity and mortality of DCM in a very important way^3, 27^.

We followed up with patients for 1080 days, and the results showed that the outcomes of male patients with DCM and patients with high NLR and CLR were adverse, consistent with a previous study^24^. Next, we comprehensively analyzed the CLR, NLR, and PLR in patients, and the results showed that cardiac function declined rapidly in female patients with a high CLR, and their prognosis was poor in the general population of DCM or in the post-PSM population. We hypothesized that in females, some immune responses are stronger than those in males, thus leading to a higher CLR in females. A high CLR could counteract the effect of other factors associated with poor prognosis in male patients with DCM. Women secrete high levels of proinflammatory cytokines, activated proinflammatory T cells, and CRP, thereby promoting inflammation^28^. In addition, an increase in the expression level of proinflammatory genes was observed in the heart muscle in females^5^. The prevalence of autoimmune diseases is higher in women, which is associated with HF development, thereby indicating differences in immunity and inflammation based on sex^29^. It has been shown that persistent low-grade chronic inflammation leads to edema and increased destruction of cardiomyocytes, which leads to increased myocardial failure and thus death. CRP, neutrophils, and lymphocytes all respond to the level of immune inflammation in the body, and their reciprocal ratios aptly respond to the state of inflammation in the body, whereas high CRP predicts the state of inflammatory activity in the body^1, 30^; thus, it has been theorized that women with a high CLR index may have a higher risk of death, and this idea is also confirmed by our large sample validation. Nevertheless, additional studies are needed to determine the underlying pathological and physiological mechanisms.

However, there are some limitations to our study. First, the sample size remains insufficient due to regional, economic, and various factors as well as challenges associated with the diagnosis of DCM, and the patients were from only two institutions. Second, due to ethnographic limitations, the included populations were all East Asian Chinese, and other races and different ethnicities have not been addressed. In addition, we included retrospective studies, which made us prone to bias in the selection of examination results. Therefore, to minimize the impact of selection bias, we selected patients based on strict inclusion and exclusion criteria to accurately reflect the actual occurrence of events. Again, our analysis of risk factors did not cover all potential factors affecting the short-term prognosis of DCM, including rapid sudden cardiac death in a short period of time and the use of device-assisted therapy. Finally, with the emergence of new antihyperlipidemic drugs (e.g., eplerenone, vericiguat, etc.), their impact on patient prognosis is unclear. Therefore, more data and long-term follow-up are needed to predict the prognosis of DCM, and we will continue to study these parameters in the future. It has been established that these immune responses promote DCM progression and can play a role in HF clinical conditions where the disease worsens. CLR is a noninvasive, inexpensive, and easily available marker for evaluating the prognosis of DCM.

## Methods

### General data

In total, 2122 patients were enrolled in the study, including 1286 patients hospitalized in the First Affiliated Hospital of Guangxi Medical University and 836 patients hospitalized in Liuzhou People’s Hospital Affiliated to Guangxi Medical University from October 2012 to May 2020 due to HF-related symptoms such as chest tightness, shortness of breath, and swelling in the lower limbs (Fig. 1).

These patients were diagnosed with DCM based on the previously described inclusion and exclusion criteria^31^. In addition, patients with a clear diagnosis of DCM included in the study based on the inclusion and exclusion criteria were informed before consenting. Finally, we included 913 patients with DCM (Fig. 1).

The patients with DCM were further evaluated using propensity score matching (PSM). The patients were followed up until their cardiac functions deteriorated. The patients were followed up until May 2023. Patients with DCM (98 males and females each) with the diagnosis were evaluated based on RWS. The initial age, weight, EF, ventricular size, etc., of male and female patients with DCM were not significantly different after propensity score matching (Fig. 1).

### Evaluating clinical and laboratory parameters

We collected the baseline clinical data of patients using electronic medical records, including patient age, sex, smoking habit (mean number of cigarettes/days > 1 and smoking continuously for > 1 year), alcohol consumption (mean intake of alcohol > 50 g/day and over 1 year), systolic blood pressure (SBP), diastolic BP (DBP), body mass index (BMI), comorbid conditions, and New York Heart Association (NYHA) grade. Additionally, laboratory tests (fasting blood glucose, glycosylated hemoglobin, complete routine, total cholesterol, triglyceride, HDL cholesterol, LDL cholesterol, N-terminal B Type I natriuretic peptide (NT-proBNP)) were conducted. The results were analyzed using an autoanalyzer (Type 7170A; Hitachi Ltd., Tokyo, Japan) at the Clinical Science Experiment Center of these hospitals. Cardiac ultrasound indicators, such as left ventricular end-diastolic diameter (LVEDD), left ventricular end-systolic diameter (LVESD), EF, and the use of therapeutic drugs, including angiotensin-converting enzyme inhibitors/angiotensin receptor antagonists, spironolactone, and β-blockers were determined. We obtained data on patients’ lifestyle habits, including smoking, alcohol consumption, and history of diseases, such as high BP, diabetes, coronary artery disease, autoimmune diseases, and radiotherapy for cancer, using medical records or questionnaires. The levels of white blood cells (WBCs), red blood cells (RBCs), neutrophils, lymphocytes, platelets (PLTs), eosinophils, and basophils were measured using an automatic blood cell analyzer (BC-6800). Next, we calculated the NLR and PLR. Furthermore, we measured the fasting glucose, CRP, IL-6, lipids in the blood, and myocardial enzyme profiles via the enzyme method using an automatic biochemical instrument. Subsequently, we calculated the CRP/lymphocyte ratio (CLR), IL-6/leukocyte ratio, and IL-6/lymphocyte ratio. Finally, we determined NT-proBNP levels using the rapid immunofluorescence method.

### Vascular measurements

We measured LVEDD, LVESD, and EF using cardiac ultrasound, i.e., echocardiography. A specialist sonographer performed all transthoracic echocardiography (TTE) evaluations, which were reviewed by a senior cardiologist. Finally, we obtained two-dimensional TTE images using a Philips IE33 ultrasound machine and evaluated them following the relevant guidelines.

### Statistical methods

We statistically analyzed the data using the SPSS 22.0 package (SPSS Inc., IL, USA). The quantitative variables were presented as the mean ± standard deviation, and qualitative variables were analyzed using the chi-squared test and presented as percentages. We measured the general characteristics of patients and controls using the unpaired Student’s t test. We determined the correlation between the risk of HF and variables as well as calculated the odds ratio (OR) and 95% confidence interval (95% CI) using unconditional logistic regression, setting HF as 1 and the control group as 0. A stepwise method was used. Propensity score matching (PSM) is commonly used in observational studies to address the imbalance of covariates between groups, and it can effectively reduce confounding bias by screening the experimental and control groups statistically to make more reasonable comparisons between the experimental and control groups and to obtain an effect similar to that of a randomized controlled study throughout the entire stage of study design^32^. Furthermore, we constructed the receiver operating characteristic (ROC) curve of variables to distinguish between different HFs. The top left corner indicates the best parameter. We calculated the area under the ROC curve (AUC) value to determine the diagnostic performance of these variables. Next, we determined the overall survival and disease-free survival in patients based on four variables using the “survival” R package. Patients were divided based on the optimal parameter of the ROC curve into four groups (high and low). Next, we plotted the Kaplan‒Meier (KM) survival curve. We constructed the nomogram using the ‘rms’ package for predicting HF risk in patients. The accuracy of the risk model in predicting HF risk was determined by discrimination using the C-statistic, and calibration was evaluated using the Hosmer‒Lemeshow χ^2^ method. The AUC value was used to determine the discriminatory ability of the risk model, and the 95% CI was calculated. The discriminative ability of the model with AUC values > 0.75 was considered relatively good. Finally, we drew the figures using R (version 3.6.0) software.

### Ethics approval and consent to participate

The authors are accountable for all aspects of the work in ensuring that questions related to the accuracy or integrity of any part of the work are appropriately investigated and resolved. The study was conducted in accordance with the Declaration of Helsinki (as revised in 2013). This study was approved by the Ethics Committee of the first affiliated Hospital of Guangxi Medical University; written informed consent was obtained from the patient himself or his close relatives.

## Data Availability

The datasets generated during and/or analysed during the current study are not publicly available due to the data belong to the hospital database but are available from the corresponding author on reasonable request.

## Abbreviations

HF: Heart failure
PSM: Propensity score matching
ROC: Receiver operating characteristic
EF: Ejection fraction
HFrEF: HF with reduced EF
HFpEF: HF with preserved EF
DCM: Dilated cardiomyopathy
RCTs: Randomized controlled trials
RWS: Real world study
BMI: Body mass index
DBP: Diastolic blood pressure
SBP: Systolic blood pressure
WBC: White blood cell counts
RBC: Red blood cell counts
PLT: Platelet counts
L: Lymphocyte
N: Neutrophil
TC: Total cholesterol
TG: Triglyceride
LDL: Low-density lipoprotein
HDL: High-density lipoprotein
CK: Creatine kinase
CK-MB: Creatine kinase isoenzyme
BNP: N-terminal B type I natriuretic peptide
LVEDD: Left ventricular end-diastolic diameter
LVESD: Left ventricular end-systolic diameter
LVEF: Left ventricular ejection fraction
CRP: C-reactive protein
NLR: Neutrophil/lymphocyte ratio
PLR: Platelet/lymphocyte ratio
CLR: CRP/lymphocyte ratio
